# The detection of canine parvovirus type 2c of Asian origin in dogs in Romania evidenced its progressive worldwide diffusion

**DOI:** 10.1186/s12917-021-02918-6

**Published:** 2021-06-05

**Authors:** Andrea Balboni, Mihaela Niculae, Serena Di Vito, Lorenza Urbani, Alessia Terrusi, Cosmin Muresan, Mara Battilani

**Affiliations:** 1 Department of Veterinary Medical Sciences, Alma Mater Studiorum - University of Bologna, Via Tolara di Sopra 50, 40064 Ozzano dell’Emilia, Bologna Italy; 2 Department of Clinical Sciences, Division of Infectious Diseases, University of Agricultural Sciences and Veterinary Medicine, Cluj-Napoca, Romania

**Keywords:** Asian CPV-2c, Canine parvovirus, Diffusion, Dog, Romania

## Abstract

**Background:**

Canine parvovirus (CPV) is one of the most important pathogens of dogs. Despite vaccination, CPV infections are still ubiquitous in dogs, and the three antigenic variants 2a, 2b and 2c are variously distributed in the canine population worldwide. To date, no information is available on CPV variants circulating in some European countries. The aim of this study was to genetically characterise the CPV detected in ten dogs with clinical signs of acute gastroenteritis in Romania. The presence of *Carnivore protoparvovirus* 1 DNA was investigated in faecal samples using an end-point PCR targeting the complete VP2 gene and positive amplicons were sequenced and analysed.

**Results:**

All ten dogs with acute gastroenteritis tested positive to *Carnivore protoparvovirus* 1 DNA in faecal samples. The identified viruses belonged to CPV-2c type, showed identical sequences of the VP2 gene and were characterised by distinctive amino acid residues in the deduced VP2 protein: 5-glicine (5Gly), 267-tirosine (267Tyr), 324-isoleucine (324Ile) and 370-arginine (370Arg). These distinctive amino acid residues have already been reported in CPV-2c widespread in Asia and occasionally detected in Italy and Nigeria.

**Conclusions:**

Since CPV-2c with VP2 amino acid residues 5Gly, 267Tyr, 324Ile and 370Arg were never reported before 2013, it can be assumed that this virus is progressively expanding its spread in the world dog population. This study adds new data about the presence of this new virus in Europe and underline worrying questions about its potential impact on the health of the canine population.

**Supplementary Information:**

The online version contains supplementary material available at 10.1186/s12917-021-02918-6.

## Background

Canine parvovirus (CPV) (family *Parvoviridae*, genus *Protoparvovirus*, species *Carnivore protoparvovirus 1*) [1] is a small, non-enveloped, single-stranded linear DNA virus of approximately 5000 nucleotides (nts) including two large open reading frames (ORFs): ORF1 encodes the two non-structural proteins NS1 and NS2, and ORF2 encodes the two structural proteins VP1 and VP2 [2]. VP2 is the major component of the icosahedrical capsid, it is involved in receptor binding and represent the most important protective antigen. Consequently, the amino acid composition of VP2 affects most of the biological characteristics of the virus.

In dogs, CPV is responsible for acute severe haemorrhagic gastroenteritis and leukopenia leading to high mortality in young puppies [3]. The original Canine parvovirus type 2 (CPV-2) was first identified in dogs in the 1970s and spread worldwide in a short time [4]. CPV-2 originated from feline panleukopenia virus (FPV) or related viruses of wild carnivores [5]. From 1979, the three antigenic variants CPV-2a, CPV-2b and CPV-2c, characterised by key amino acid substitutions in the VP2 protein [6, 7], gradually replaced the original CPV-2 type, which is currently no longer widespread in nature but contained only in some commercial vaccines. The CPV antigenic variants replicate and spread more effectively in susceptible hosts, gaining the ability to infect cats [8, 9]. The detection of other amino acid changes in the VP2 protein and the poor phylogenetic resolution supporting the three variants are leading to a progressive move away from the use of the CPV-2a, CPV-2b and CPV-2c terminology towards the use of the defining amino acid substitutions for each of these antigenic variants [10]. Nevertheless, references to the traditional nomenclature are prevalent in the literature and adopted herein.

Despite vaccination, CPV infections are still ubiquitous in dogs, frequently as result of immunisation failure and prolonged environmental persistence. The three antigenic variants 2a, 2b and 2c are variously distributed in the canine population worldwide and, normally, more than one variant coexist in the same geographical area [11]. In Europe, CPV-2a is predominant in most countries, exception for Ireland and the UK where CPV-2b is the most prevalent variant; also CPV-2c was found in European countries [12], and it has been reported to predominate in some areas such as in Portugal, Poland and Southern Italy (Sicily region) [13–16].

To date, no information is available on CPV variants circulating in some European countries. The aim of this study was to genetically characterise the CPV detected in dogs with clinical signs of acute gastroenteritis in Romania.

## Results

**Fig. 1:**
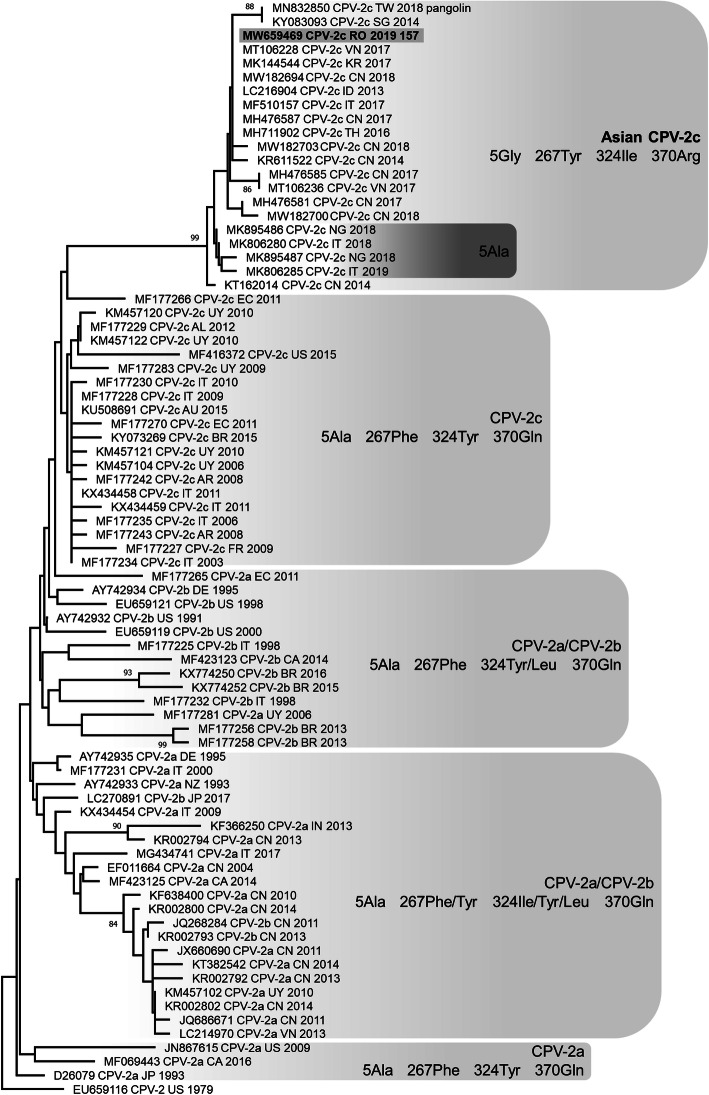
Phylogenetic tree constructed on the complete VP2 nucleotide sequences of canine parvovirus. Phylogenetic tree based on the complete VP2 gene nucleotide sequences of canine parvovirus (CPV) obtained in this study and reference strains in the GenBank database (see Additional file 1). Phylogeny was constructed by MEGA X version 10.1.7. using Neighbor-Joining method and the Tamura 3-parameters (T92) model with gamma distribution. Statistical support was provided by bootstrapping with 1000 replicates. Bootstrap values greater than 80% are indicated on the respective branches. Highlighted: Sequences generated in this study (only sequence MW659469_CPV-2c_RO_2019_157 is reported because all the sequences obtained are identical). The antigenic variants and the amino acid residues in positions 5, 267, 324 and 370 are reported for each cluster

All ten dogs with acute gastroenteritis tested positive to *Carnivore protoparvovirus* 1 DNA in faecal samples and were included in the study (lab ID numbers from 157 to 166, Table 1). Seven out of 10 dogs were described with incomplete vaccination protocols involving one single administration of an old type CPV-2-based vaccine in the first weeks of life, using a modified live CPV-2 vaccine or an inactivated CPV-2 vaccine (Table 1). The complete nucleotide sequence of VP2 gene was obtained for all the viruses identified and were identical. Based on the critical amino acid residues of the deduced VP2 protein, the viruses identified were classified as CPV and belonged to the 2c variant, owing to the occurrence of the amino acid glutamate in position 426 (codon GAA) [6]. BLAST analysis allowed to identify 32 reference sequences of CPV-2c with full query coverage and complete nucleotide identity (Additional file 1) and several other CPV-2c reference sequences showing nucleotide identity ≥99.89%, reported from 2013 to 2020 in several Asian regions, Italy and Nigeria [17–25]. A nucleotide identity of 99.89% was also calculated between our sequences and a CPV-2c strain detected in a pangolin (*Manis pentadactyla pentadactyla*) in Taiwan in 2018 (MN832850) [26]. All these reference CPV-2c and the CPV-2c identified in Romania in this study were characterised by distinctive amino acid residues in the deduced VP2 protein: 5-glicine (5Gly), 267-tirosine (267Tyr), 324-isoleucine (324Ile) and 370-arginine (370Arg), with the exception of some CPV-2c reported in Italy and Nigeria that showed the most common 5-alanine (5Ala) residue [20, 22]. Phylogenetic tree showed a monophyletic cluster, supported by a high bootstrap value, which groups the CPV-2c characterised by the VP2 amino acid residues 5Gly(Ala), 267Tyr, 324Ile and 370Arg, including the viral sequences identified in the Romanian dogs in this study, separated from other CPV-2c (Fig. 1).

**Table 1:** Signalment data and vaccination status of the dogs tested positive for CPV DNA

Dogs	Date of sampling	Breed	Sex	Age (months)	Geographical origin	Vaccination^a^
157	28/04/2019	Mixed breed	M	6	Cluj-Napoca (RO)	No
158	16/08/2019	Mixed breed	F	7	Cluj-Napoca (RO)	Yes (A)
159	10/07/2019	Mixed breed	F	6	Cluj-Napoca (RO)	Yes (A)
160	23/08/2019	Mixed breed	F	6	Cluj-Napoca (RO)	Yes (A)
161	03/08/2019	German shepherd	F	3	Cluj-Napoca (RO)	Yes (A)
162	28/07/2019	Mixed breed	M	72	Cluj-Napoca (RO)	Yes (A)
163	13/07/2019	Jack Russell terrier	M	2	Cluj-Napoca (RO)	No
164	26/07/2019	Siberian husky	F	4	Cluj-Napoca (RO)	Yes (A)
165	27/11/2019	Mixed breed	M	2	Cluj-Napoca (RO)	No
166	29/11/2019	American Staffordshire terrier	F	5	Cluj-Napoca (RO)	Yes (B)

^a^ Dogs undergone only one administration of old type CPV-2-based vaccine in the first weeks of life: A) modified live CPV-2 vaccine or B) inactivated CPV-2 vaccine

F: female. M: male. RO: Romania

## Discussion

This study reports the first genetic characterisation of CPV identified in dog with acute gastroenteritis in Romania. Although a small number of samples were analysed in this study precluding firm conclusions on the epidemiology of CPV molecular types in Romania, all the viruses identified belonged to CPV-2c variant, had identical VP2 gene nucleotide sequences, and showed distinctive amino acid residues in the deduced VP2 protein: 5Gly, 267Tyr, 324Ile and 370Arg. These distinctive amino acid residues have already been reported in CPV-2c widespread in Asia and occasionally detected in Italy and Nigeria [18–23, 25]. The close correlation between these CPV-2c is also evidenced by the phylogenetic analysis of the VP2 gene. CPV-2c with these typical amino acid residues in the deduced VP2 protein are previously recognised as “Asian CPV-2c” because detected for the first time in Asia [18, 24]. The “Asian CPV-2c” has progressively become the dominant strain in several areas of Asia within a few years from its first identification [18], and at the same time it has also reached Europe and Africa [19, 20, 22]. Many of the mutations observed in CPV are short-lived and both parallel evolution and reversion are commonplace for this virus [10]. However, as CPV-2c with VP2 amino acid residues 5Gly, 267Tyr, 324Ile and 370Arg was never reported before 2013 and it has been frequently detected since then, it can be assumed that a virus with this distinctive amino acid profile is progressively expanding its spread in the world dog population, continuing to acquire mutations such as the amino acid change Ala→Gly in residue 5 [20, 22]. Other Authors have also hypothesised that CPV-2c containing these four amino acid substitutions is more virulent compared with previous viruses [23]. Further studies are needed to understand if the amino acid residues that characterise the “Asian CPV-2c” group are able to determine some advantage in the virus-host interaction, in terms of replication, pathogenesis, spread, or elusion of the immune response.

CPV-2c was reported in association with severe disease in dogs vaccinated with old type CPV-2-based vaccine [27]. Also in this study, “Asian CPV-2c” DNA was identified in seven dogs with acute gastroenteritis who received one administration of a CPV-2-based vaccine. Several causes may have led to the immunisation failure: i) first of all, the vaccination protocol incompletely performed compared to WSAVA vaccination guidelines group that recommend CPV vaccination at 6–8 weeks of age, then every 2–4 weeks until 16 weeks of age or older to avoid the interfering titres of maternally-derived antibodies [28]; ii) the use of an inactivated vaccine (in one dog, lab ID 166, Table 1), that has lower immunogenicity than a modified live vaccine; iii) a pre-exposure to CPV prior to vaccination. Furthermore, the role of CPV variants in causing vaccination failure is debated. Several studies have demonstrated that currently available vaccines, including those prepared with the original CPV-2, confer a good degree of protection against CPV-2a, CPV-2b and CPV-2c [29]. Previous studies raised concerns regarding the efficacy of vaccines currently adopted for prophylaxis in dogs in providing full protection against the “Asian CPV-2c” [17, 23]. In this regard, some studies suggest greater efficacy of modified live CPV-2b vaccines against the CPV-2c variant [30, 31]. Future challenge studies should be of fundamental importance to evaluate the ability of currently adopted vaccines to prevent clinical manifestation and reduce the spread of this new virus.

## Conclusions

This study adds new data on the progressive spread of “Asian CPV-2c” with VP2 amino acid residues 5Gly, 267Tyr, 324Ile and 370Arg in Europe and raises concerns regarding the potential impact this new virus may have on the health of the canine population.

## Methods

### Study design and samples

In this study, the presence of *Carnivore protoparvovirus* 1 DNA was investigated in faecal samples of ten dogs affected by acute gastroenteritis and tested positive to CPV antigen by using a rapid in-clinic test (two dogs were tested positive with the Rapid CPV Ag test kit, VetExpert, Łomianki, Poland and eight dogs with the Rapid CPV Ag Test kit, Bionote, Hwaseong-si, South Korea) in a private animal clinic (Professionalvet) or in the Department of Clinical Sciences, University of Agricultural Sciences and Veterinary Medicine (USAMV), Cluj-Napoca, Romania, from April 2019 to November 2019. Dogs tested positive to *Carnivore protoparvovirus* 1 DNA were included in the study and the identified viruses were genetically characterised by sequencing and analysis of the VP2 gene.

### Molecular detection of CPV DNA

DNA extraction from faeces was carried out by using the NucleoSpin Tissue Kit (Macherey-Nagel, Düren, Germany) according to the manufacturer’s instructions. Extracted DNA was stored at − 20 °C until use. The presence of *Carnivore protoparvovirus* 1 DNA was investigated using an end-point polymerase chain reaction (PCR) targeting a fragment of 1887 nts, comprising the complete VP2 gene (1755 nts), with the primers VP2_2684-2705_For (5′- ACC AAC TAA AAG AAG TAA ACC A − 3′) and VP2_4544-4570_Rev (5′- GTA ATA AAC ATA AAA ACA TAG TAA GTA − 3′). A proofreading DNA polymerase (Phusion Hot Start II High-Fidelity DNA Polymerase, Thermo Fisher Scientific, Life Technologies, Waltham, MA, USA) was used. The reactions were performed in a total volume of 50 μL containing 0.5 μM of each primer, 5X HF buffer, 2.5 mM dNTP, 2 U/μL Phusion Hot Start II DNA Polymerase and 5 μL of DNA extract. The thermal cycling consisted of an initial denaturation at 98 °C for 30 s followed by 35 cycles of denaturation at 98 °C for 10 s, annealing at 55.6 °C for 30 s and elongation at 72 °C for 1 min, followed by a final elongation step at 72 °C for 10 min. A DNA extract of FPV positive sample was used as positive control (lab ID 1033/2009) [32]. A no template control, consisting of ultrapure water, underwent analysis simultaneously. PCR products (5 μL) were separated by electrophoresis in a 1.0% agarose gel in TAE buffer and visualized by UV light after staining with Midori Green Advance DNA Stain (Nippon Genetics, Düren, Germany). Amplicons of the expected size were considered positive.

### Sequence analysis

Amplicons of the expected size were purified using the QIAquick PCR Purification Kit (Qiagen, Hilden, Germany) according to the manufacturer’s instructions and directly sequenced by Sanger method (BioFab Research, Rome, Italy) using both forward and reverse primers, and a third internal primer, primer 41 (5′- GCC CTT GTG TAG ACG C -3′) [33].

Complete VP2 gene sequences were assembled, analysed with BLAST web interface (https://blast.ncbi.nlm.nih.gov/Blast.cgi, word size 16, accessed March 12, 2021), aligned with 78 reference sequences of CPV from GenBank database (https://www.ncbi.nlm.nih.gov/genbank/, Additional file 2) using the ClustalW method implemented in BioEdit 7.2.5 and translated into amino acid sequences. Phylogeny was carried out on complete VP2 nucleotide sequences using the software MEGA X version 10.1.7 [34]. Phylogenetic tree was constructed using Neighbor-Joining method and the Tamura 3-parameters model with gamma distribution. The robustness of individual nodes on the phylogenetic tree was estimated using 1000 bootstrap replicates and bootstrap values > 80 were indicated at the corresponding node.

The complete VP2 gene sequences obtained in this study are openly available in INSDC database (http://www.insdc.org/; ID: MW659469- MW659476).

## Supplementary Information

**Additional file 1.** BLAST web interface (https://blast.ncbi.nlm.nih.gov/Blast.cgi, word size 16, accessed March 12, 2021) analysis result: 32 reference sequences of CPV-2c showing full query coverage and complete nucleotide identity with the complete viral VP2 gene sequences obtained in this study. CN: China. ID: Indonesia. IT: Italy. KR: South Korea. NG: Nigeria. TH: Thailand. VN: Vietnam.

**Additional file 2.** Canine parvovirus (CPV) nucleotide sequences obtained in this study and reference strains used for analysis. In bold: CPV nucleotide sequences obtained in this study. AL: Albania. AR: Argentina. AU: Australia. BR: Brazil. CA: Canada. CN: China. DE: Germany. EC: Ecuador. FR: France. ID: Indonesia. IN: India. IT: Italy. JP: Japan. KR: South Korea. NG: Nigeria. NZ: New Zeeland. RO: Romania. SG: Singapore. TH: Thailand. TW: Taiwan. US: United States of America. UY: Uruguay. VN: Vietnam.

## Data Availability

The datasets supporting the conclusions of this article are included within the article (and its additional files). The complete VP2 gene sequences obtained in this study are openly available in INSDC database (http://www.insdc.org/; ID: MW659469- MW659476).

## Abbreviations

Ala: alanine
Arg: arginine
CPV: canine parvovirus
CPV-2: original “old type” canine parvovirus type 2
CPV-2a: canine parvovirus type 2a
CPV-2b: canine parvovirus type 2b
CPV-2c: canine parvovirus type 2c
FPV: feline panleukopenia virus
Gln: glutamine
Gly: glycine
Ile: isoleucine
Leu: leucine
NS1: non-structural protein 1
NS2: non-structural protein 2
nts: nucleotides
ORF: open reading frames
PCR: polymerase chain reaction
Phe: phenylalanine
Tyr: tirosine
USAMV: University of Agricultural Sciences and Veterinary Medicine
VP1: viral protein 1
VP2: viral protein 2
WSAVA: World Small Animal Veterinary Association
