# Supplementary material for: The detection of canine parvovirus type 2c of Asian origin in dogs in Romania evidenced its progressive worldwide diffusion
Source: BMC Vet Res. 2021 Jun 5;17:206. doi: 10.1186/s12917-021-02918-6 (PMC8180150; doi:10.1186/s12917-021-02918-6)
Supplement: Supplementary file 1 — Additional file 1. BLAST web interface (https://blast.ncbi.nlm.nih.gov/Blast.cgi, word size 16, accessed March 12, 2021) analysis result: 32 reference sequences of CPV-2c showing full query coverage and complete nucleotide identity with the complete viral VP2 gene sequences obtained in this study. CN: China. ID: Indonesia. IT: Italy. KR: South Korea. NG: Nigeria. TH: Thailand. VN: Vietnam. [file 12917_2021_2918_MOESM1_ESM.docx]

**BLAST web interface (**[**https://blast.ncbi.nlm.nih.gov/Blast.cgi**](https://blast.ncbi.nlm.nih.gov/Blast.cgi)**, word size 16, accessed March 12, 2021) analysis result: 32 reference sequences of CPV-2c showing full query coverage and complete nucleotide identity with the complete viral VP2 gene sequences obtained in this study**

| **GenBank ID** | **Variant** | **Origin** | **Year** |
| --- | --- | --- | --- |
| LC216904 | CPV-2c | ID | 2013 |
| MF510157 | CPV-2c | IT | 2017 |
| MG013488 | CPV-2c | CN | 2017 |
| MH476583 | CPV-2c | CN | 2017 |
| MH476587 | CPV-2c | CN | 2017 |
| MH711894 | CPV-2c | TH | 2016 |
| MH711902 | CPV-2c | TH | 2016 |
| MK144544 | CPV-2c | KR | 2017 |
| MN451678 | CPV-2c | NG | 2018 |
| MN451682 | CPV-2c | NG | 2018 |
| MN810876 | CPV-2c | CN | 2017-2019 |
| MN810878 | CPV-2c | CN | 2017-2019 |
| MN810879 | CPV-2c | CN | 2017-2019 |
| MN810889 | CPV-2c | CN | 2017-2019 |
| MN810890 | CPV-2c | CN | 2017-2019 |
| MN810893 | CPV-2c | CN | 2017-2019 |
| MN810896 | CPV-2c | CN | 2017-2019 |
| MN810904 | CPV-2c | CN | 2017-2019 |
| MN810909 | CPV-2c | CN | 2017-2019 |
| MN810910 | CPV-2c | CN | 2017-2019 |
| MN810912 | CPV-2c | CN | 2017-2019 |
| MT106228 | CPV-2c | VN | 2017 |
| MT179767 | CPV-2c | CN | 2019 |
| MW182694 | CPV-2c | CN | 2018 |
| MW182698 | CPV-2c | CN | 2018 |
| MW182706 | CPV-2c | CN | 2019 |
| MW182709 | CPV-2c | CN | 2019 |
| MW182712 | CPV-2c | CN | 2018 |
| MW182714 | CPV-2c | CN | 2018 |
| MW182715 | CPV-2c | CN | 2018 |
| MW182719 | CPV-2c | CN | 2020 |
| MW182720 | CPV-2c | CN | 2020 |

CN: China. ID: Indonesia. IT: Italy. KR: South Korea. NG: Nigeria. TH: Thailand. VN: Vietnam.
