# Supplementary material for: The detection of canine parvovirus type 2c of Asian origin in dogs in Romania evidenced its progressive worldwide diffusion
Source: BMC Vet Res. 2021 Jun 5;17:206. doi: 10.1186/s12917-021-02918-6 (PMC8180150; doi:10.1186/s12917-021-02918-6)
Supplement: Supplementary file 2 — Additional file 2. Canine parvovirus (CPV) nucleotide sequences obtained in this study and reference strains used for analysis. In bold: CPV nucleotide sequences obtained in this study. AL: Albania. AR: Argentina. AU: Australia. BR: Brazil. CA: Canada. CN: China. DE: Germany. EC: Ecuador. FR: France. ID: Indonesia. IN: India. IT: Italy. JP: Japan. KR: South Korea. NG: Nigeria. NZ: New Zeeland. RO: Romania. SG: Singapore. TH: Thailand. TW: Taiwan. US: United States of America. UY: Uruguay. VN: Vietnam. [file 12917_2021_2918_MOESM2_ESM.docx]

**Canine parvovirus (CPV) nucleotide sequences obtained in this study and reference strains used for analysis**

| **GenBank ID** | **Variant** | **Origin** | **Year** | **Note** |
| --- | --- | --- | --- | --- |
| AY742932 | CPV-2b | US | 1991 |  |
| AY742933 | CPV-2a | NZ | 1993 |  |
| AY742934 | CPV-2b | DE | 1995 |  |
| AY742935 | CPV-2a | DE | 1995 |  |
| D26079 | CPV-2a | JP | 1993 |  |
| EF011664 | CPV-2a | CN | 2004 |  |
| EU659116 | CPV-2 | US | 1979 |  |
| EU659119 | CPV-2b | US | 2000 |  |
| EU659121 | CPV-2b | US | 1998 |  |
| JN867615 | CPV-2a | US | 2009 |  |
| JQ268284 | CPV-2b | CN | 2011 |  |
| JQ686671 | CPV-2a | CN | 2011 |  |
| JX660690 | CPV-2a | CN | 2011 |  |
| KF366250 | CPV-2a | IN | 2013 |  |
| KF638400 | CPV-2a | CN | 2010 |  |
| KM457102 | CPV-2a | UY | 2010 |  |
| KM457104 | CPV-2c | UY | 2006 |  |
| KM457120 | CPV-2c | UY | 2010 |  |
| KM457121 | CPV-2c | UY | 2010 |  |
| KM457122 | CPV-2c | UY | 2010 |  |
| KR002792 | CPV-2a | CN | 2013 |  |
| KR002793 | CPV-2b | CN | 2013 |  |
| KR002794 | CPV-2a | CN | 2013 |  |
| KR002800 | CPV-2a | CN | 2014 |  |
| KR002802 | CPV-2a | CN | 2014 |  |
| KR611522 | CPV-2c | CN | 2014 |  |
| KT162014 | CPV-2c | CN | 2014 |  |
| KT382542 | CPV-2a | CN | 2014 |  |
| KU508691 | CPV-2c | AU | 2015 |  |
| KX434454 | CPV-2a | IT | 2009 |  |
| KX434458 | CPV-2c | IT | 2011 |  |
| KX434459 | CPV-2c | IT | 2011 |  |
| KX774250 | CPV-2b | BR | 2016 |  |
| KX774252 | CPV-2b | BR | 2015 |  |
| KY073269 | CPV-2c | BR | 2015 |  |
| KY083093 | CPV-2c | SG | 2014 |  |
| LC214970 | CPV-2a | VN | 2013 |  |
| LC216904 | CPV-2c | ID | 2013 |  |
| LC270891 | CPV-2b | JP | 2017 |  |
| MF069443 | CPV-2a | CA | 2016 |  |
| MF177225 | CPV-2b | IT | 1998 |  |
| MF177227 | CPV-2c | FR | 2009 |  |
| MF177228 | CPV-2c | IT | 2009 |  |
| MF177229 | CPV-2c | AL | 2012 |  |
| MF177230 | CPV-2c | IT | 2010 |  |
| MF177231 | CPV-2a | IT | 2000 |  |
| MF177232 | CPV-2b | IT | 1998 |  |
| MF177234 | CPV-2c | IT | 2003 |  |
| MF177235 | CPV-2c | IT | 2006 |  |
| MF177242 | CPV-2c | AR | 2008 |  |
| MF177243 | CPV-2c | AR | 2008 |  |
| MF177256 | CPV-2b | BR | 2013 |  |
| MF177258 | CPV-2b | BR | 2013 |  |
| MF177265 | CPV-2a | EC | 2011 |  |
| MF177266 | CPV-2c | EC | 2011 |  |
| MF177270 | CPV-2c | EC | 2011 |  |
| MF177281 | CPV-2a | UY | 2006 |  |
| MF177283 | CPV-2c | UY | 2009 |  |
| MF416372 | CPV-2c | US | 2015 |  |
| MF423123 | CPV-2b | CA | 2014 |  |
| MF423125 | CPV-2a | CA | 2014 |  |
| MF510157 | CPV-2c | IT | 2017 |  |
| MG434741 | CPV-2a | IT | 2017 |  |
| MH476581 | CPV-2c | CN | 2017 |  |
| MH476585 | CPV-2c | CN | 2017 |  |
| MH476587 | CPV-2c | CN | 2017 |  |
| MH711902 | CPV-2c | TH | 2016 |  |
| MK144544 | CPV-2c | KR | 2017 |  |
| MK806280 | CPV-2c | IT | 2018 |  |
| MK806285 | CPV-2c | IT | 2019 |  |
| MK895486 | CPV-2c | NG | 2018 |  |
| MK895487 | CPV-2c | NG | 2018 |  |
| MN832850 | CPV-2c | TW | 2018 | Host: pangolin |
| MT106228 | CPV-2c | VN | 2017 |  |
| MT106236 | CPV-2c | VN | 2017 |  |
| MW182694 | CPV-2c | CN | 2018 |  |
| MW182700 | CPV-2c | CN | 2018 |  |
| MW182703 | CPV-2c | CN | 2018 |  |
| **MW659469** | **CPV-2c** | **RO** | **2019** | **Lab ID: 157** |
| **MW659470** | **CPV-2c** | **RO** | **2019** | **Lab ID: 158** |
| **MW659471** | **CPV-2c** | **RO** | **2019** | **Lab ID: 159** |
| **MW659472** | **CPV-2c** | **RO** | **2019** | **Lab ID: 160** |
| **MW659473** | **CPV-2c** | **RO** | **2019** | **Lab ID: 161** |
| **MW659474** | **CPV-2c** | **RO** | **2019** | **Lab ID: 162** |
| **MW659475** | **CPV-2c** | **RO** | **2019** | **Lab ID: 163** |
| **MW659476** | **CPV-2c** | **RO** | **2019** | **Lab ID: 164** |
| **MW659477** | **CPV-2c** | **RO** | **2019** | **Lab ID: 165** |
| **MW659478** | **CPV-2c** | **RO** | **2019** | **Lab ID: 166** |

In bold: CPV nucleotide sequences obtained in this study.

AL: Albania. AR: Argentina. AU: Australia. BR: Brazil. CA: Canada. CN: China. DE: Germany. EC: Ecuador. FR: France. ID: Indonesia. IN: India. IT: Italy. JP: Japan. KR: South Korea. NG: Nigeria. NZ: New Zeeland. RO: Romania. SG: Singapore. TH: Thailand. TW: Taiwan. US: United States of America. UY: Uruguay. VN: Vietnam.
